# Chronic environmental circadian disruption increases atherosclerosis and dyslipidemia in female, but not male, *ApolipoproteinE*-deficient mice

**DOI:** 10.3389/fphys.2023.1167858

**Published:** 2023-03-29

**Authors:** Jeffrey M. Chalfant, Deborah A. Howatt, Victoria B. Johnson, Lisa R. Tannock, Alan Daugherty, Julie S. Pendergast

**Affiliations:** ^1^ Department of Biology, University of Kentucky, Lexington, KY, United States; ^2^ Saha Cardiovascular Research Center, University of Kentucky, Lexington, KY, United States; ^3^ Department of Veterans Affairs, Lexington, KY, United States; ^4^ Department of Internal Medicine, University of Kentucky, Lexington, KY, United States; ^5^ Barnstable Brown Diabetes Center, University of Kentucky, Lexington, KY, United States; ^6^ Department of Physiology, University of Kentucky, Lexington, KY, United States

**Keywords:** circadian rhythm, shift work, cardiovascular disease, eating rhythm, fasting

## Abstract

Shift work chronically disrupts circadian rhythms and increases the risk of developing cardiovascular disease. However, the mechanisms linking shift work and cardiovascular disease are largely unknown. The goal of this study was to investigate the effects of chronically shifting the light-dark (LD) cycle, which models the disordered exposure to light that may occur during shift work, on atherosclerosis. Atherosclerosis is the progressive accumulation of lipid-filled lesions within the artery wall and is the leading cause of cardiovascular disease. We studied *ApolipoproteinE*-deficient (*ApoE* ^*−/−*^) mice that are a well-established model of atherosclerosis. Male and female *ApoE* ^*−/−*^ mice were housed in control 12L:12D or chronic LD shift conditions for 12 weeks and fed low-fat diet. In the chronic LD shift condition, the light-dark cycle was advanced by 6 h every week. We found that chronic LD shifts exacerbated atherosclerosis in female, but not male, *ApoE* ^*−/−*^ mice. In females, chronic LD shifts increased total serum cholesterol concentrations with increased atherogenic VLDL/LDL particles. Chronic LD shifts did not affect food intake, activity, or body weight in male or female *ApoE* ^*−/−*^ mice. We also examined eating behavior in female *ApoE* ^*−/−*^ mice since aberrant meal timing has been linked to atherosclerosis. The phases of eating behavior rhythms, like locomotor activity rhythms, gradually shifted to the new LD cycle each week in the chronic LD shift group, but there was no effect of the LD shift on the amplitudes of the eating rhythms. Moreover, the duration of fasting intervals was not different in control 12L:12D compared to chronic LD shift conditions. Together these data demonstrate that female *ApoE* ^*−/−*^ mice have increased atherosclerosis when exposed to chronic LD shifts due to increased VLDL/LDL cholesterol, independent of changes in energy balance or feeding-fasting cycles.

## 1 Introduction

The 24-h cycle of light and dark is a distinctive feature of living on Earth. It is so pervasive that nearly every animal has evolved an internal timekeeping mechanism, the circadian system, to coordinate ∼24-h internal rhythms of behavior (e.g., eating and locomotion) and physiology (e.g., metabolic cycles) with the light-dark cycle (Pittendrigh, 1993). However, modern lifestyles disrupt this fine tuning of internal rhythms with external cycles. More than 20 million Americans work night or rotating shifts, which chronically disrupts their circadian rhythms (McMenamin, 2007). Shift workers experience abnormal exposure to light and have disrupted eating and activity/sleeping patterns (Geliebter et al., 2000; de Assis et al., 2003; Bank et al., 2015; Molzof et al., 2017; Molzof et al., 2022). Critically, disruption of the circadian system with shift work is associated with obesity, metabolic syndrome, and cardiovascular disease (CVD) (Kawachi et al., 1995; Boggild and Knutsson, 1999; Sookoian et al., 2007; Vyas et al., 2012; Jankowiak et al., 2016; Torquati et al., 2018).

The mammalian circadian system is comprised of clocks in nearly every tissue in the body that are organized hierarchically (Damiola et al., 2000; Yamazaki et al., 2000; Yoo et al., 2004). The main clock, the suprachiasmatic nucleus (SCN) in the hypothalamus, receives information about the environmental light-dark cycle from the retina and then coordinates the phases of peripheral clocks located in body tissues (Moore and Eichler, 1972; Stephan and Zucker, 1972; Yamazaki et al., 2000; Yoo et al., 2004). In this way, each tissue is fine-tuned to regulate its specific functions at the proper time of day relative to other tissues and relative to the environmental light-dark cycle. Previous studies demonstrated that mutant mice that had non-functional molecular circadian rhythms (e.g., *Bmal1* KO and *Clock* ^*Δ19*^ mutant mice) had increased pathological vascular remodeling and atherosclerosis [(Anea et al., 2009; Ma et al., 2013; Pan et al., 2013; Pan et al., 2016; Huo et al., 2017; Xie et al., 2020; Lin et al., 2022; Shen et al., 2022), but see (Yang et al., 2016)]. However, these mutant mice do not recapitulate circadian disruption that commonly occurs in humans, which is often environmentally induced.

Several prior studies used an environmental light trigger of circadian disruption to study atherosclerosis in mice. These studies used various lighting cycles, including inverting the light-dark cycle, advancing the light-dark cycle by 8 h, or constant light, which resulted in increased atherosclerosis in mice (Zhu et al., 2016; Schilperoort et al., 2020a; Schilperoort et al., 2020b; Xie et al., 2020; Figueiro et al., 2021). However, the mice in these prior studies were fed Western diet, which independently causes metabolic dysfunction, obesity, and disrupts circadian rhythms (Kohsaka et al., 2007; Hatori et al., 2012; Eckel-Mahan et al., 2013; Pendergast et al., 2013; Inoue et al., 2021). We took a unique approach and fed mice low-fat diet to avoid the confounding effects of Western diet. We found that chronic exposure to constant lighting conditions, which caused severe circadian disruption, increased atherosclerosis and VLDL/LDL-cholesterol concentrations in male, but not female, *ApoE* ^*−/−*^ mice fed low-fat diet (Chalfant et al., 2020).

The goal of the current study was to use a light-induced model of circadian disruption, that better approximates the disruption experienced by shift workers than constant light, in mice fed low-fat diet, to study the link between circadian disruption and atherosclerosis. Aberrant meal timing is associated with cardiovascular disease and has been proposed as a mechanism by which shift work could exacerbate atherosclerosis (St-Onge et al., 2017; Jordan et al., 2019; Manoogian et al., 2022). Our model using low-fat diet feeding allowed us to investigate the possible role of aberrant eating rhythms as a mechanism linking light-induced circadian disruption and atherosclerosis.

## 2 Materials and methods

### 2.1 Animals

*ApoE* ^*−/−*^ mice were purchased from The Jackson Laboratory (stock #002052) on the C57BL/6J background (N10) and then backcrossed for 3 additional generations with C57BL/6J mice. *ApoE* ^*−/−*^ mice used for experiments were therefore N13 C57BL/6J and were generated from crosses of *ApoE* ^*+/−*^ mice. All mice were bred in our vivarium in 12L:12D with food (Teklad 2918 irradiated) and water available *ad libitum*. Pups were weaned at 3 weeks old, group-housed, and genotyped according to The Jackson Laboratory protocol. All procedures were approved by the University of Kentucky Institutional Animal Care and Use Committee (protocols 2015-2211 and 2021-3842).

### 2.2 Experimental protocol

At 7 weeks old, male and female *ApoE* ^*−/−*^ mice were single-housed in cages (33 cm × 17 cm × 14 cm) with locked running wheels (wheels could not rotate) in light-tight boxes in 12L:12D (white LEDs, light intensity 250-350 lux). Mice were fed a low-fat diet (10% kcal fat; 3.85 kcal/g, Research Diets D12450K) and water *ad libitum* for the entire experiment. Mice were randomized to either the control or chronic LD shift group. Mice in the control group were housed in the same 12L:12D cycle for 14 weeks (lights on 06:00 to 18:00). In the chronic shift condition, the mice were housed in standard 12L:12D for 2 weeks, and then the light-dark cycle was advanced by 6 h each week for 12 weeks. We advanced, rather than delayed, the light-dark cycle because it takes longer for mice to resynchronize to phase advances than delays, which most likely causes more severe circadian disruption (Zee et al., 1992; Davidson et al., 2006). Body weight and food intake were measured weekly during the 3 h before lights out at Zeitgeber time (ZT) 9-12, where ZT0 is lights on and ZT12 is lights out. At the conclusion of the experiment, mice were anesthetized by inhaled isoflurane and then euthanized by cervical dislocation at ZT6-9. Cardiac puncture was used to collect blood. Aortas were collected as described below. One female mouse in the control 12L:12D group was excluded due to gross organ abnormality (enlarged kidney).

### 2.3 Aorta collection and analysis

Aortas were collected and analyzed as described previously (Daugherty and Whitman, 2003). Briefly, aortas were perfused with 0.9% NaCl (wt/vol) by left cardiac puncture, dissected from the root to the iliac bifurcation, and then stored in 10% neutrally-buffered formalin for 24-h. Atherosclerotic lesion areas were measured in *en face* aortas, defined as the ascending arch to 3 mm distal to the root of the left subclavian artery, in which the aorta was cut longitudinally and imaged with Image-Pro 7.0 software. Lesion areas were analyzed by two researchers (one was blinded to the experimental condition and the other was not).

### 2.4 Analysis of lipids

After anesthesia, blood was collected by cardiac puncture and centrifuged to isolate serum. Total serum cholesterol (Cholesterol E Enzymatic Kit, Wako Pure Chemical Industries kits Mountain View, CA) and triglycerides concentrations (L-Type Triglyceride M Enzyme Color A and Color B, Wako Pure Chemical Industries kits Mountain View, CA) were measured with commercially available kits. Individual 50 µl serum samples were fractioned by fast protein liquid chromatography (FPLC) using a Superose 6 column. Cholesterol and triglyceride concentrations were measured in each fraction with the kits above. Lipoprotein distributions were analyzed in 4-5 samples selected to be around the mean total serum cholesterol concentration.

### 2.5 Analysis of locomotor activity

General locomotor activity was continuously measured using passive infrared sensors (Adafruit, New York) interfaced to ClockLab software (Actimetrics Inc., Wilmette, IL). Total locomotor activity was determined for each mouse by summing the activity counts for the entire 14-week experiment. Actograms were plotted in 6-min bins using the normalized format.

### 2.6 Analysis of eating behavior

Eating behavior was continuously monitored for 2 weeks. At 7 weeks old, female *ApoE* ^*−/−*^ mice were housed in light-tight boxes in control 12L:12D for 1 week and then in the chronic LD shift condition for 1 week. Lighting conditions, cages, and low-fat diet were identical to the long experiment described above. Eating behavior was recorded using infrared video cameras (HD 48LED 940 nm CMOS 800TVL IR-CUT Dome camera waterproof IF CCTV) connected to a Lorex DVR system (MPX HD 1080p Security System DVR). Eating behavior data were analyzed as previously characterized, and included mice eating food from the feeder or in their paws (Pendergast et al., 2013). Eating behavior was measured in 1-min bins.

Two parameters of eating behavior were analyzed. The first eating behavior parameter analyzed was the daily rhythm of eating behavior. Each of the 5 days in control 12L:12D and the 5 days after the LD shift were analyzed. Twenty-four hours of data (from lights on) were plotted in circular histograms using Oriana 4.0 software. Circular statistics were used to determine the vector of each day of eating behavior. The vector length and direction were defined as the amplitude and phase of the eating behavior rhythm, respectively. The second eating parameter analyzed was the length of fasting intervals. Fasting intervals were measured during 5 continuous days of control 12L:12D and then for 5 days after the shift of the LD cycle. A fasting interval was defined as the length of time between meal offset and the successive meal onset. A meal was defined as ≥10 min of eating in 30 min (eating did not have to be continuous).

### 2.7 Statistical analyses

Atherosclerosis lesion area, total serum cholesterol and triglyceride concentrations, cumulative food intake, and cumulative locomotor activity in *ApoE* ^*−/−*^ mice of the same sex were compared between control 12L:12D and chronic LD shifts using independent two-tailed Student’s *t*-tests, unless the data were not normally distributed or had unequal variance, in which case the Mann-Whitney test was used. The study was designed to analyze females and males as separate cohorts, so we report only sex-specific effects herein. The duration and number of fasting intervals and amplitudes of daily rhythms of eating behavior were compared in female *ApoE* ^*−/−*^ mice that were first in control 12L:12D and then the LD shift condition, so paired Student’s tests were used, unless the data were not normally distributed, in which case, Paired Sample Wilcoxon Signed Rank tests were used. Statistical tests were performed with OriginPro 2017 (Northampton, MA). Data are presented as the mean ± SEM. Significance was ascribed at *p* < 0.05.

## 3 Results

### 3.1 Chronic shifts of the light-dark cycle exacerbate atherosclerosis and dyslipidemia in female, but not male, *ApoE* ^*−/−*^ mice

**FIGURE 1:**
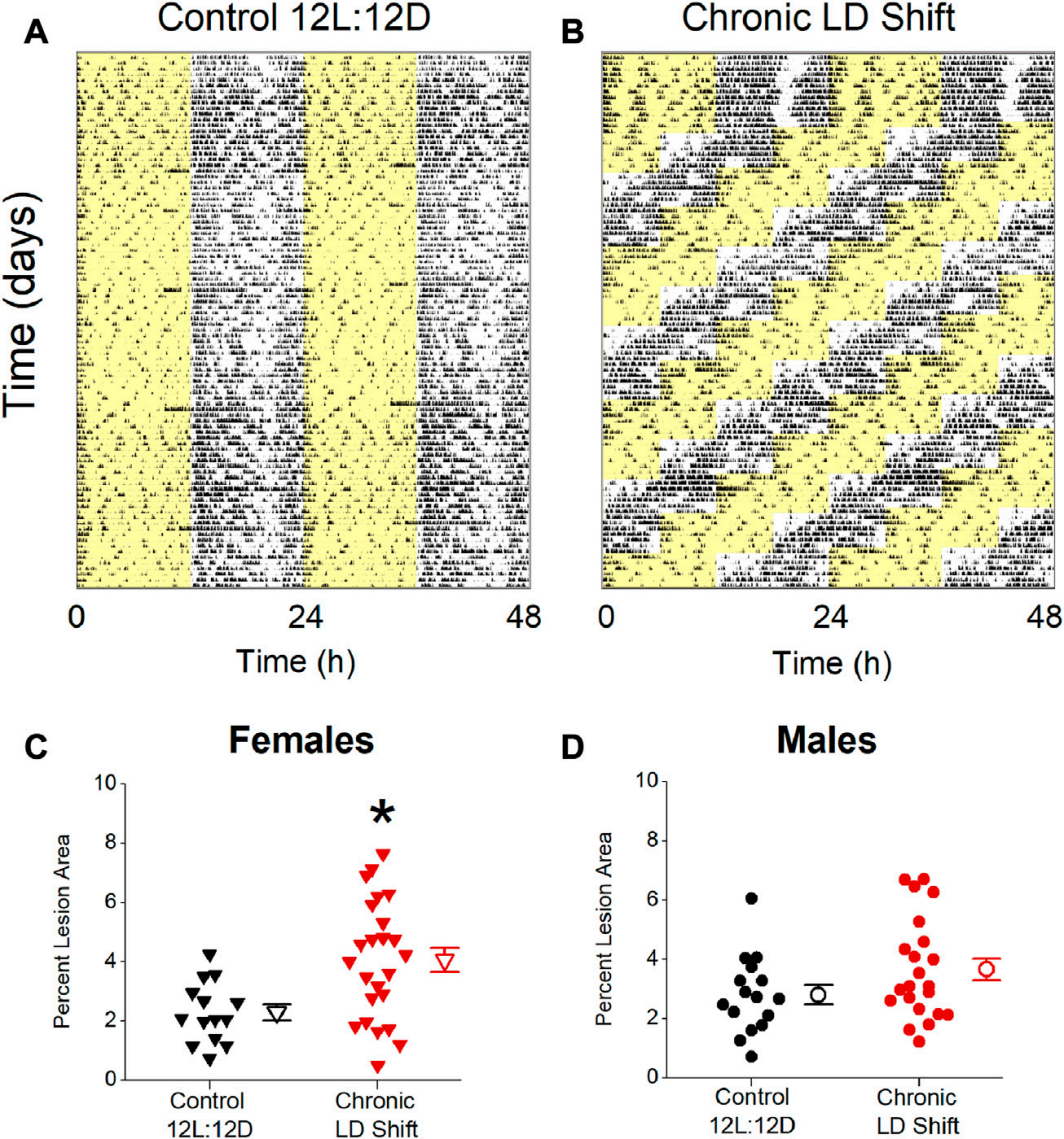
Chronic LD shifts increased atherosclerosis in female, but not male, *ApoE* ^*−/−*^ mice. Representative actograms of general locomotor activity recorded from *ApoE* ^*−/−*^ mice housed in control 12L:12D **(A)** or chronic LD shift (b, time of lights off advanced 6 h each week) conditions and fed low-fat diet. Yellow shading shows lights on. Percent atherosclerotic lesion area in female **(C)** and male **(D)** *ApoE* ^*−/−*^ mice housed in control 12L:12D (black symbols) or chronic LD shifts (red symbols). **(C,D)** individual mice are closed symbols and open symbols are mean ± SEM. *p = 0.01.

We first investigated the effects of chronic shifts of the light-dark cycle on the sizes of atherosclerosis lesions in male and female *ApoE* ^*−/−*^ mice. A control group of *ApoE* ^*−/−*^ mice was housed in a standard light/dark cycle (Figure 1A, 12 h of light/12 h of dark: Control 12L:12D), and the experimental group of *ApoE* ^*−/−*^ mice were exposed to the chronic LD shift protocol for 12 weeks (Figure 1B: Chronic LD shift). In this protocol, the light-dark cycle was advanced by 6 h every week. In the chronic LD shift protocol, the onsets of activity rhythms in male and female mice gradually advanced until they were aligned with the shifted LD cycle (Supplementary Figure S1). Female *ApoE* ^*−/−*^ mice advanced to the new LD cycle faster than male *ApoE* ^*−/−*^ mice (Supplementary Figure S1). The mice were fed a 10% kcal low-fat diet for the duration of the experiment to avoid the confounding effects of obesity, insulin resistance, and circadian disruption (e.g., disruption of the eating rhythm) that are caused by feeding high fat/high cholesterol diets (Kohsaka et al., 2007; Hatori et al., 2012; Pendergast et al., 2013). We found that chronic LD shifts increased atherosclerosis in female (Figure 1C; Table 1, Mann-Whitney *p* = 0.01), but not male (Figure 1D; Table 1; *t*-test *p* = 0.10), *ApoE* ^*−/−*^ mice.

**TABLE 1:** Descriptive statistics and statistical analyses of atherosclerosis and lipids. Comparisons were made within the same sex for each parameter; *Tests were two-tailed.

	Control 12L:12D mean ± SEM (n)	Chronic LD shift mean ± SEM (n)	Test*	Test statistic, *p*-value
*En face* lesion area (% of arch)				
Females	2.29 ± 0.27 (14)	4.06 ± 0.41 (24)	Mann-Whitney	U = −2.59, *p* = 0.01
Males	2.80 ± 0.32 (16)	3.65 ± 0.36 (22)	*t*-test	*t*_*36*_ *=* -1.68, *p* = 0.10
Total serum cholesterol (mg/dl)				
Females	426.0 ± 19.4 (14)	495.0 ± 14.4 (24)	*t*-test	*t*_*36*_ = −2.88,*p* = 0.007
Males	622.0 ± 37.0 (16)	614.4 ± 27.8 (22)	*t*-test	*t*_*36*_ = 0.17, *p* = 0.87
Total serum triglycerides (mg/dl)				
Females	31.1 ± 4.5 (11)	40.0 ± 2.7 (21)	*t*-test	*t*_*30*_ = −1.82, *p* = 0.08
Males	70.0 ± 8.6 9)	79.4 ± 11.3 (21)	Mann-Whitney	U = 91, *p* = 0.89

**FIGURE 2:**
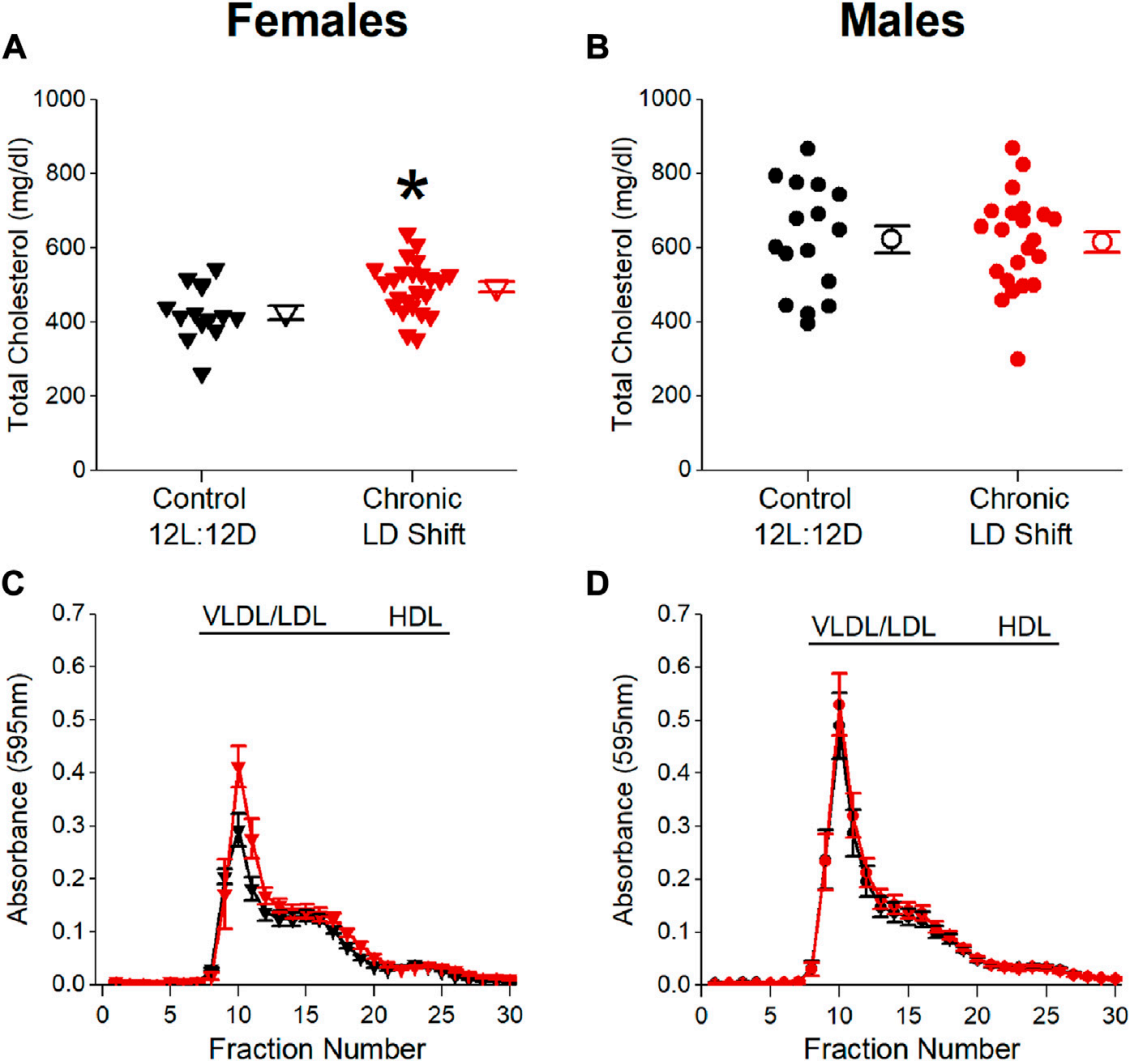
Chronic LD shifts increased dyslipidemia in female, but not male, *ApoE* ^*−/−*^ mice. Total serum cholesterol concentrations **(A,B)** and distribution of lipoprotein-cholesterol (c, d; FPLC was performed in n = 4–5/group) in female **(A,C)** and male **(B,D)** *ApoE* ^*−/−*^ mice housed in control 12L:12D (black symbols) or chronic LD shifts (red symbols). **(A,B)**: individual mice are closed symbols and open symbols are mean ± SEM. *p = 0.007.

We next investigated possible factors by which chronic LD shifts could increase atherosclerosis in female *ApoE* ^*−/−*^ mice. Female *ApoE* ^*−/−*^ mice in the chronic LD shift condition had increased total serum cholesterol concentrations (Figure 2A; Table 1, Mann-Whitney *p* = 0.007), due to increased VLDL/LDL particles (Figure 2C), compared to females in control 12L:12D. There were no significant differences in total serum cholesterol concentrations (Figure 2B; Table 1, *t*-test *p* = 0.87) or in the distribution of cholesterol on lipoproteins (Figure 2D) in male *ApoE* ^*−/−*^ mice housed in control 12L:12D compared to chronic LD shifts. Chronic LD shifts did not significantly alter total serum triglycerides concentrations in female (Table 1, *t*-test *p* = 0.08) or male *ApoE* ^*−/−*^ mice (Table 1, Mann-Whitney *p* = 0.89).

### 3.2 Chronic LD shifts did not alter energy balance in female and male *ApoE* ^*−/−*^ mice

**FIGURE 3:**
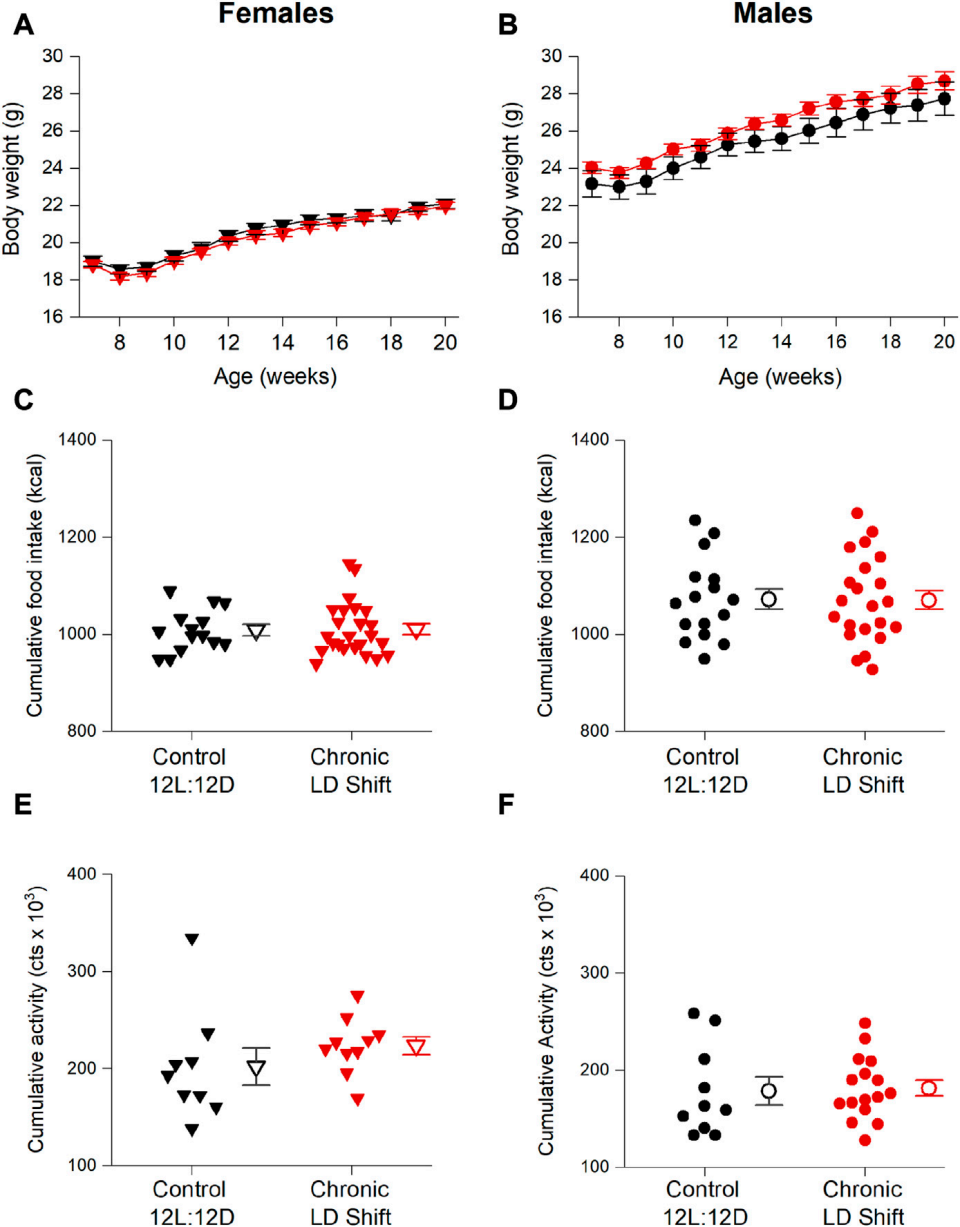
Chronic LD shifts did not alter energy balance in male and female *ApoE* ^*−/−*^ mice. Female **(A,C,E)** and male **(B,D,F)** *ApoE−/−* mice were maintained in control 12L:12D (black symbols) or chronic LD shifts (red symbols) for 12 weeks. Body weights **(A,B)** mean ± SEM) were measured weekly, and cumulative food intake **(C,D)** and cumulative activity counts **(E,F)** were summed for the entire experiment. **(C–F)**: individual mice are closed symbols and open symbols are mean ± SEM.

We next determined whether energy balance was altered by the chronic LD shift condition (Figure 3). Chronic LD shifts did not affect body weights of female (Figure 3A; Table 2) or male (Figure 3B; Table 2) *ApoE* ^*−/−*^ mice in chronic LD shifts compared to the control 12L:12D condition. Cumulative calorie consumption was not significantly altered by chronic LD shifts in female (Figure 3C; Table 2, *t*-test *p* = 0.91) or male (Figure 3D; Table 2, *t*-test *p* = 0.94) *ApoE* ^*−/−*^ mice. Likewise, cumulative locomotor activity during the experiments did not significantly differ between lighting conditions in female (Figure 3E; Table 2, *t*-test *p* = 0.31) or male (Figure 3F; Table 2, *t*-test *p* = 0.84) *ApoE* ^*−/−*^ mice. Together these results demonstrate that chronic LD shifts did not affect food intake, activity, or body weight in *ApoE* ^*−/−*^ mice.

**TABLE 2:** Descriptive statistics and statistical analyses of metabolic parameters. Comparisons were made within the same sex for each parameter; *Tests were two-tailed; ^Some mice removed from the activity analysis due to faulty infrared sensors.

Metabolic parameter	Control 12L:12D mean ± SEM (n)	Chronic LD shift mean ± SEM (n)	Test*	Test statistic, *p*-value
Body weight (beginning, g)				
Females	19.0 ± 1.1 (14)	18.8 ± 0.9 (24)	*t*-test	*t*_*36*_ = 0.55, *p* = 0.59
Males	23.2 ± 0.7 (16)	24.0 ± 0.3 (23)	*t*-test	*t*_*36*_ = −1.23, *p* = 0.23
Body weight (ending, g)				
Females	22.1 ± 0.9 (14)	22.0 ± 0.9 (24)	*t*-test	*t*_*33*_ = 0.37, *p* = 0.71
Males	27.7 ± 0.9 (16)	28.7 ± 0.5 (22)	*t*-test	*t*_*36*_ = −1.00, *p* = 0.32
Cumulative food intake (kcal)				
Females	1009 ± 12 (14)	1011 ± 11 (24)	*t-*test	*t*_*36*_ = −0.11, *p* = 0.91
Males	1072 ± 21 (16)	1070 ± 19 (22)	*t-*test	*t*_*36*_ = 0.07*, p =* 0.94
Cumulative activity (x10^3^ ct)				
Females	202.2 ± 19.2 (9^)	223.7 ± 9.1 (10^)	*t-*test	*t*_*17*_ = −1.05, *p* = 0.31
Males	178.6 ± 14.8 (10^)	181.6 ± 8.1 (16^)	*t-*test	*t* ^*24*^ = −0.20, *p* = 0.84

### 3.3 Chronic LD shifts did not affect eating behavior in female *ApoE* ^*−/−*^ mice

**FIGURE 4:**
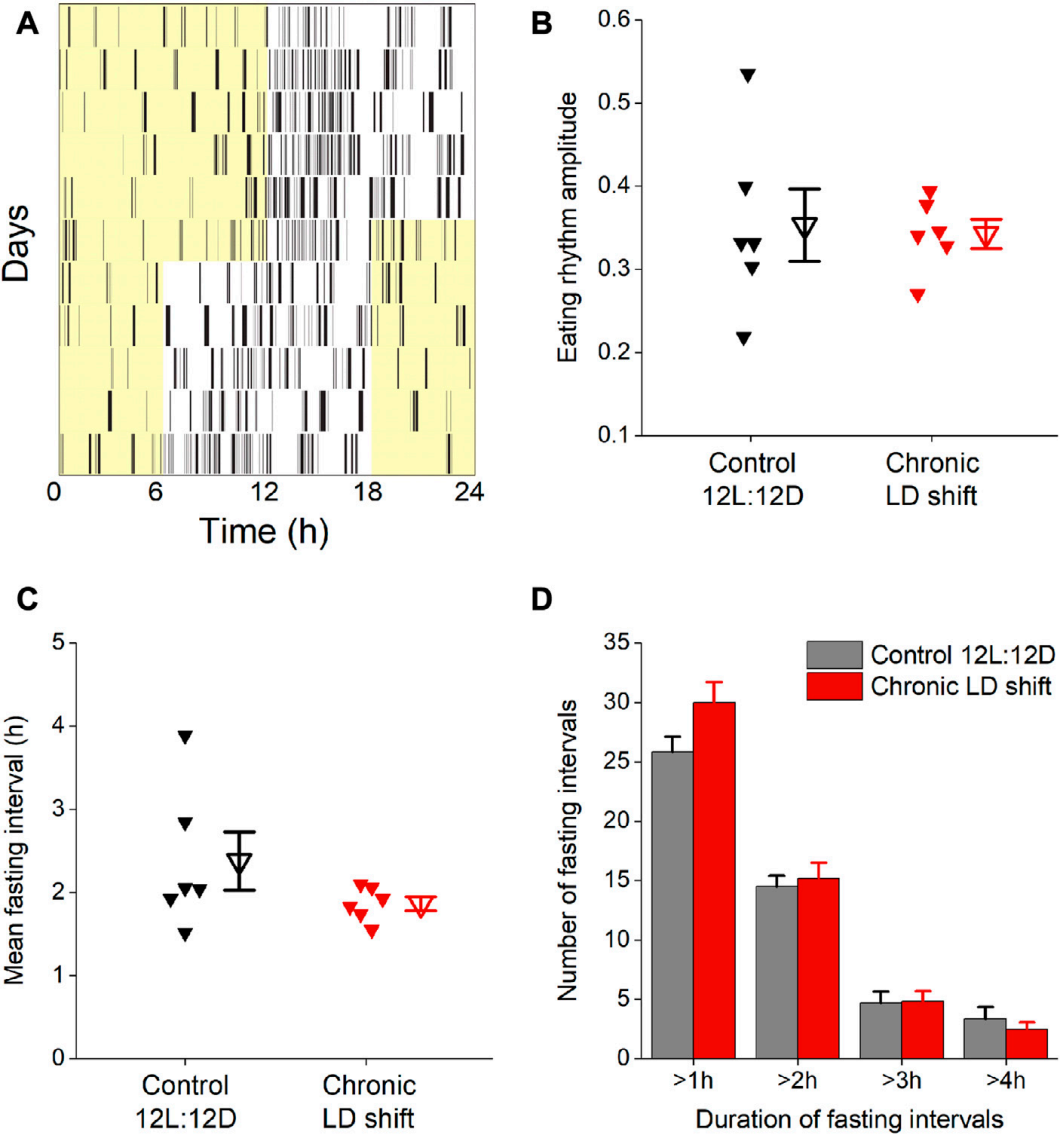
Chronic LD shifts did not affect eating behavior in female *ApoE* ^*−/−*^ mice. Eating behavior was measured with infrared video cameras during control 12L:12D and when the LD cycle was advanced 6 h **(A)**. The amplitudes of the daily rhythms of eating behavior **(B)**, duration of daily fasting intervals **(C)**, and number of fasting intervals that were >1 h, >2 h, >3 h, and >4 h **(D)** were measured. b,c: individual mice are closed symbols and open symbols are mean ± SEM.

We next determined whether meal timing and meal patterning were disrupted by chronic LD shifts in female *ApoE* ^*−/−*^ mice (Figure 4). We measured daily rhythms of eating behavior to assess meal timing. In control 12L:12D, female *ApoE* ^*−/−*^ mice had robust daily rhythms of nocturnal eating behavior such that they ate mostly during the dark phase, but had several meals during their inactive phase (Figure 4A), which is similar to meal timing in wild-type mice (Pendergast et al., 2013; Omotola et al., 2019). When the LD cycle was advanced by 6h, the eating behavior rhythm gradually shifted to the new LD cycle but retained high-amplitude rhythms during the shift (Figures 4A, B; Supplementary Figures S2, S3). There was no significant effect of the LD shift on the amplitude of the eating behavior rhythm (Figure 4B; Table 3, *t*-test *p* = 0.83).

**TABLE 3:** Descriptive statistics and statistical analyses of eating behavior in female *ApoE* ^*−/−*^ mice. *Paired Student’s *t*-tests were two-tailed for data that were normally distributed and had equal variances, while Paired Sample Wilcoxon Signed Rank tests were used if data were not normally distributed or did not have equal variances, n = 6.

Eating behavior parameter	Control 12L:12D mean ± SEM	Chronic LD shift mean ± SEM	Test*	Test statistic, *p*-value
Amplitude of daily rhythm (r)	0.35 ± 0.04	0.34 ± 0.02	*t*-test	*t*_*5*_ = 0.23, *p* = 0.83
Duration of fasting intervals (h)	2.4 ± 0.3	1.9 ± 0.1	Wilcoxon	Z = 0.84, *p =* 0.40
Number of fasting intervals				
>1 h	25.8 ± 1.3	30.0 ± 1.7	*t*-test	*t*_*10*_ = −1.81, *p* = 0.13
>2 h	14.5 ± 0.9	15.2 ± 1.4	Wilcoxon	Z = −0.21, *p =* 0.83
>3 h	4.7 ± 1.0	4.8 ± 0.9	*t*-test	*t*_*10*_ = −0.20, *p* = 0.85
>4 h	3.3 ± 1.1	2.5 ± 0.6	*t*-test	*t*_*10*_ = 1.05, *p* = 0.34

We also measured the effect of shifting the LD cycle on fasting intervals. There was no effect of the LD shift on the duration of fasting intervals in female *ApoE* ^*−/−*^ mice (Figure 4C; Table 3, Wilcoxon *p* = 0.4). We also analyzed the number of short- and long-duration fasting intervals. There was no significant difference in the number of short- or long-duration fasting intervals in control 12L:12D compared to the LD shift condition (Figure 4D; Table 3).

## 4 Discussion

Our goal was to study atherosclerosis in mice under conditions that mimic severe light-induced circadian disruption that also occurs during shift work. We chose to study atherosclerosis because it is the primary cause of coronary artery disease which is the most common type of heart disease in men and women in the U.S. We used *Apolipoprotein E* ^*−/−*^ (*ApoE* ^*−/−*^) mice because, like humans, they spontaneously develop atherosclerosis, whereas other murine models need additional stimuli to induce atherosclerosis development (von Scheidt et al., 2017). For example, many murine atherosclerosis studies feed mice an atherogenic Western diet which induces obesity and insulin resistance and could therefore confound data interpretation because obesity and insulin resistance are independent risk factors for atherosclerosis (King et al., 2010). Our goal was also to study the role of eating behavior in regulating atherosclerosis, and high-fat diets are known to disrupt eating rhythms, even in control 12L:12D, which would add another variable to our study (Kohsaka et al., 2007; Pendergast et al., 2013). Thus, we chose to study mice fed low-fat diet to isolate the effect of chronic LD shifts on atherosclerosis.

In this study, we advanced the light-dark cycle by 6 h every week. This protocol is commonly used in studies that seek to disrupt circadian rhythms in rodent models, and has been shown previously to increase mortality, pathological immune responses, and kidney injury in mice and rats (Davidson et al., 2006; Castanon-Cervantes et al., 2010; Adams et al., 2013; Hill et al., 2021). Consistent with prior studies in wild-type rodents, we found that the onset of daily activity in *ApoE* ^*−/−*^ mice advanced gradually after the light-dark cycle was advanced. The onset of activity was aligned with the new LD cycle about 6 days after the shift, at which time the LD cycle was advanced again. We also found that the eating behavior rhythm gradually advanced and was realigned with the new LD cycle 6 days after the shift. Thus, both eating and activity rhythms were constantly shifting to the new LD cycle for the 12 weeks of the experiment.

We found that chronic LD shifts increased atherosclerosis and pathogenic VLDL/LDL-cholesterol in female mice. Our findings are consistent with prior studies that inverting the LD cycle increased atherosclerosis in female APOE*3-Leiden.CETP and low-density lipoprotein receptor knockout mice (Schilperoort et al., 2020a; Figueiro et al., 2021). One study showed that light-induced circadian disruption also increased atherosclerosis in male *ApoE* ^*-/*^ mice (Xie et al., 2020). A major difference between these prior studies and our study is that the mice were fed Western diet (high in fat and cholesterol) in previous studies while we fed mice low-fat diet. Western diet has pathological effects on lipids, glucose homeostasis, and energy balance. In our study, there was no effect of chronic LD shifts on body weight, food intake, or activity levels. Thus, circadian disruption increased atherosclerosis and pathogenic VLDL/LDL-cholesterol in females independent of obesity. Low-fat diet feeding in our study also revealed sex-specific effects of chronic LD shifts on atherosclerosis. Western diet feeding in the prior studies may have masked the sex-specific effects of light-induced circadian disruption on atherosclerosis because it is known that diets high in fat differentially affect circadian rhythms in male and female mice. High-fat diet feeding disrupts eating and tissue rhythms in male mice, but not in gonadally intact female mice (Kohsaka et al., 2007; Pendergast et al., 2013; Palmisano et al., 2017; Omotola et al., 2019). Our experimental design removed the confounding effects of diet on both metabolism and rhythms and isolated the effects of light-induced circadian disruption on lipids and atherosclerosis. This may be why we observed a sex-specific increase in atherosclerosis in female, but not male, *ApoE* ^*−/−*^ mice.

We found that chronic LD shifts exacerbated atherosclerosis in female, but not male *ApoE* ^*−/−*^ mice. Many clinical epidemiological studies that found an association between circadian disruption and CVD were also in women, specifically in female nurses which is a commonly studied shift work population (Kawachi et al., 1995; Vetter et al., 2016; Jorgensen et al., 2017). The sex-specific effects of chronic LD shifts on atherosclerosis in females and males cannot be attributed to cholesterol concentrations since in our experiment *ApoE* ^*−/−*^ males had higher concentrations of total and VLDL/LDL-cholesterol than females. Moreover, the sex-specific effect may be a special feature of circadian disruption that causes misalignment of behavioral rhythms and the LD cycle since in our previous study we found that *ApoE* ^*−/−*^ males, but not females, had increased atherosclerosis in a constant lighting condition, which causes more severe circadian disruption (some mice became arrhythmic) than chronic LD shifts (Chalfant et al., 2020). Future studies will investigate whether sex hormones differentially regulate lipids in misaligned (chronic LD shifts) and arrhythmic (LL) circadian disruption conditions.

Epidemiological and clinical studies have shown that aberrant meal timing (e.g., skipping breakfast and eating at night) increases the risk for CVD [reviewed in (St-Onge et al., 2017)]. Shift workers have disrupted rhythms of food intake, including consuming more calories at night and snacking throughout the night, compared to day workers, and this aberrant meal timing has been linked to CVD risk factors (Lennernas et al., 1995; de Assis et al., 2003; Heath et al., 2012; Bank et al., 2015; Molzof et al., 2017; Molzof et al., 2022). Likewise, studies in mice have shown that mistimed feeding, or eating during the inactive phase, caused metabolic dysfunction (Arble et al., 2009; Bray et al., 2012). Additionally, time-restricted feeding interventions that provided food only at the correct time of day, during the active phase, inhibited diet-induced obesity and dyslipidemia (Hatori et al., 2012; Manoogian et al., 2022). Overall, these prior studies in mice and humans have suggested that a robust, high-amplitude eating rhythm, with eating consolidated during the active phase, is effective at improving risk factors for CVD. We found that female *ApoE* ^*−/−*^ mice maintained high-amplitude daily rhythms of eating behavior during the shift of the LD cycle that did not differ from those in control 12L:12D. The phase, or timing, of the eating behavior rhythm gradually shifted to the new LD cycle, but most eating was consolidated and aligned with activity. These data suggest that activity and eating are not misaligned during LD shifts and that eating is still consolidated in a normal time window, at least in mice.

It is also possible that changes in the feeding-fasting cycle could contribute to atherosclerosis during chronic LD shifts. Daily fasting reduces pro-inflammatory monocytes in circulation, thus reductions in fasting intervals could underlie the development of atherosclerotic lesions in mice in chronic LD shift conditions (Jordan et al., 2019). However, we found no evidence that the duration or incidence of fasting intervals was affected by LD shifts. Thus, changes in the feeding-fasting cycle are not likely to be a mechanism by which chronic LD shifts increase atherosclerosis.

Sleep disruption is also associated with atherosclerosis in epidemiological studies (Cappuccio et al., 2011). Moreover, sleep fragmentation increased inflammatory monocytes and atherosclerosis in mice (McAlpine et al., 2019). The chronic LD shift protocol we used in this study was shown previously to cause 10% reduction in sleep in C57BL/6J mice, but this sleep loss did not exacerbate inflammation (Brager et al., 2013). While we cannot rule out sleep disruption as a mechanism contributing to atherosclerosis in our model, it is unlikely that sleep loss had a marked contribution to the atherosclerosis in this study.

In sum, our study shows that chronic LD shifts exacerbate atherosclerosis and dyslipidemia in female mice, independent of changes in energy balance or meal patterning and eating rhythms. It is known that chronic LD shifts cause misalignment of circadian clocks in tissues. Specifically, the main clock in the SCN shifts its phase to the new LD cycle faster than the liver clock shifts. This could lead to misalignment of lipid processing by the liver and dyslipidemia that ultimately causes atherosclerosis. Future studies should examine the role of the misaligned liver clock in regulating inflammation and lipid processing during chronic LD shifts.

## Data Availability

The original contributions presented in the study are included in the article/Supplementary Material, further inquiries can be directed to the corresponding author.
