# Supplementary material for: Chronic environmental circadian disruption increases atherosclerosis and dyslipidemia in female, but not male, ApolipoproteinE-deficient mice
Source: Front Physiol. 2023 Mar 29;14:1167858. doi: 10.3389/fphys.2023.1167858 (PMC10090465; doi:10.3389/fphys.2023.1167858)
Supplement: Supplementary file 1 [file Image1.pdf]

Supplementary Material

Chronic Environmental Circadian Disruption Increases Atherosclerosis and Dyslipidemia in Female, but not Male, *ApolipoproteinE*-deficient Mice

Jeffrey M. Chalfant, Deborah A. Howatt, Victoria B. Johnson, Lisa R. Tannock, Alan Daugherty, Julie S. Pendergast*

*** Correspondence:** julie.pendergast@uky.edu

Supplementary Figures and Tables

1.1 Supplementary Figures

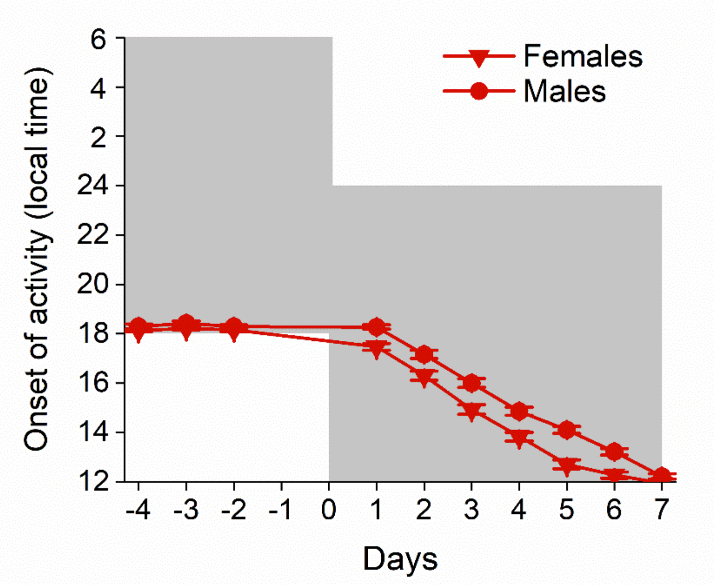

Figure S1. Female *ApoE*$^{-/-}$ mice reentrain to the shifted LD cycle faster than male ApoE$^{-/-}$ mice. The locomotor activity rhythm gradually shifts to the advanced LD cycle in female (triangles) and male (circles) *ApoE*$^{-/-}$ mice. Females have earlier onsets of activity than males on days 1-7 after the LD shift (Two-way RM ANOVA sex*day $F=7.8$, $p=0.003$). Data are mean±SEM.

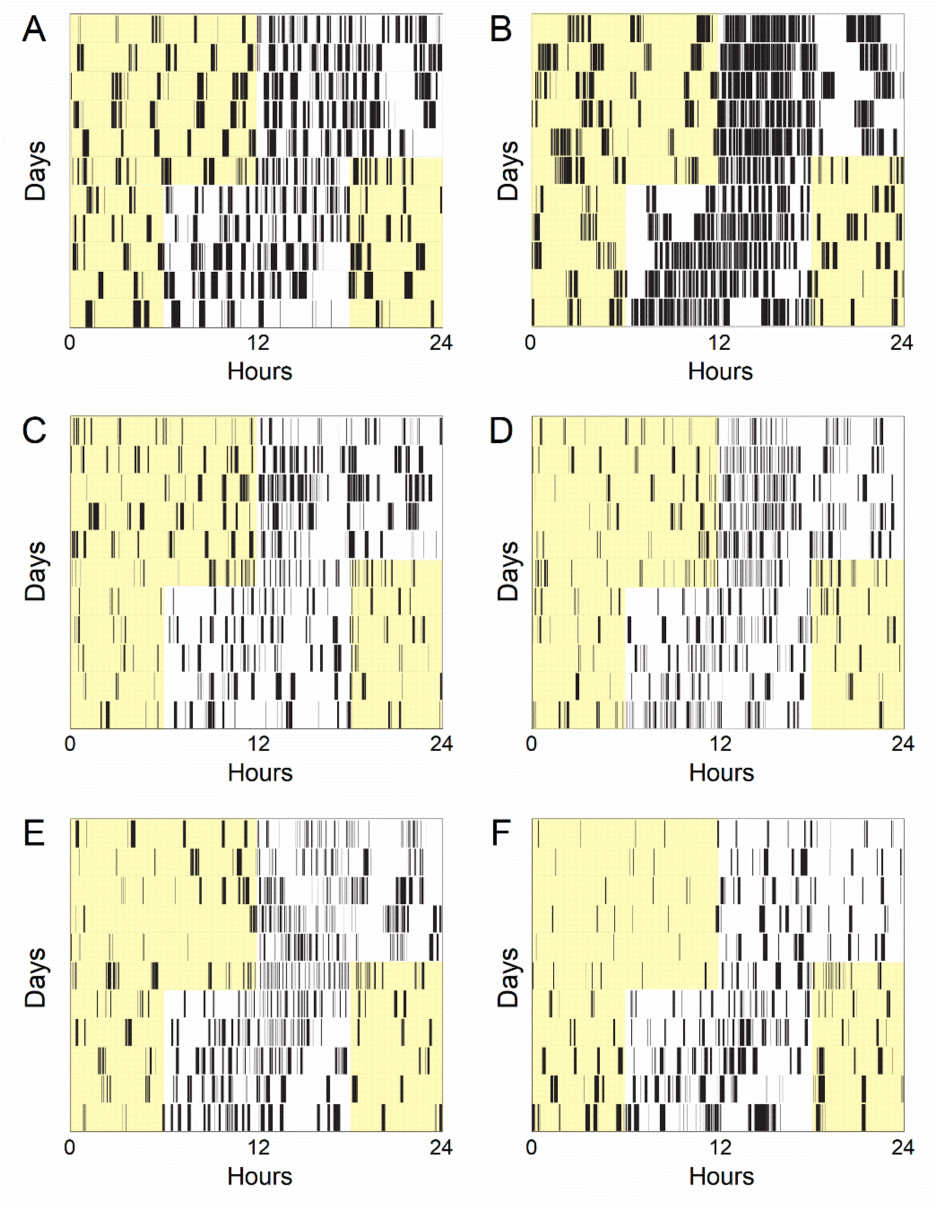

Figure S2. Eating behavior in female *ApoE*$^{-/-}$ mice during a shift of the LD cycle. Actograms of eating behavior (in 1-min bins) are shown for individual female *ApoE*$^{-/-}$ mice (a-f). Lights on is shown by yellow shading.

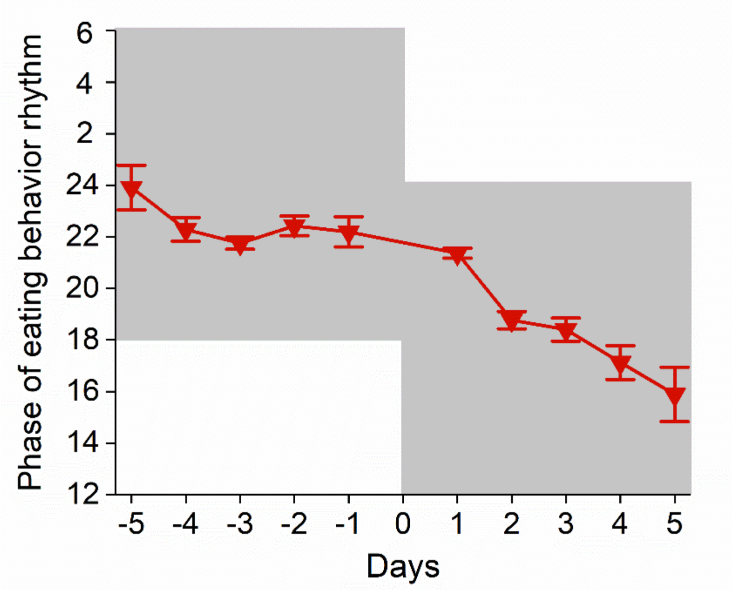

Figure S3. Reentrainment of the eating behavior rhythm during the LD shift. The locomotor activity rhythm gradually shifts to the advanced LD cycle in female *ApoE*$^{-/-}$ mice. Data are mean±SEM.
